# Supplementary material for: IFRA: A Machine Learning-Based Instrumented Fall Risk Assessment Scale Derived from an Instrumented Timed Up and Go Test in Stroke Patients
Source: Healthcare (Basel). 2026 Jan 16;14(2):228. doi: 10.3390/healthcare14020228 (PMC12841214; doi:10.3390/healthcare14020228)
Supplement: Supplementary file 1 [file healthcare-14-00228-s001.zip › healthcare-4010194-supplementary.pdf]

Supplementary Materials

S1 List of features extrapolated from data acquired through IMU

Table S1: Full list of features obtained from processing the inertial data, split according to the constituent phases of the TUG test. For each feature, the corresponding unit of measurement is reported in square brackets, if applicable.

Instrumented TUG Features
Total Duration [s]
Sit-to-Walk Duration [s]
180° Turn Duration [s]
Turn Duration in the Turn-to-Sit Phase [s]
Turn-to-Sit Duration [s]
Walk/Turn Ratio Outward
Walk/Turn Ratio Return
Walk/Turn Ratio Overall
Walk Duration including the 180° Turn [s]
Total Number of Steps
Range Anterior-Posterior Acceleration during the Sit-to-Walk Transition [m/s^2]
Range Medio-Lateral Acceleration during the Sit-to-Walk Transition [m/s^2]
Range Vertical Acceleration during the Sit-to-Walk Transition [m/s^2]
Root Mean Square of the Anterior-Posterior Acceleration during the Sit-to-Walk Transition [m/s^2]
Root Mean Square of the Medio-Lateral Acceleration during the Sit-to-Walk Transition [m/s^2]
Root Mean Square of the Vertical Acceleration during the Sit-to-Walk Transition [m/s^2]
Jerk Score Anterior-Posterior Acceleration during the Sit-to-Walk Transition [m]
Jerk Score Medio-Lateral Acceleration during the Sit-to-Walk Transition [m]
Jerk Score Vertical Acceleration during the Sit-to-Walk Transition [m]
Range of the Angular Velocity about Anterior-Posterior Axis during the Sit-to-Walk Transition [°/s]
Range of the Angular Velocity about Medio-Lateral Axis during the Sit-to-Walk Transition [°/s]
Range of the Angular Velocity about Vertical Axis during the Sit-to-Walk Transition [°/s]
Root Mean Square of the Angular Velocity about Anterior-Posterior Axis during the Sit-to-Walk Transition [°/s]
Root Mean Square of the Angular Velocity about Medio-Lateral Axis during the Sit-to-Walk Transition [°/s]
Root Mean Square of the Angular Velocity about Vertical Axis during the Sit-to-Walk Transition [°/s]
Normalised Jerk Score of the Angular Velocity about Anterior-Posterior Axis during the Sit-to-Walk Transition
Normalised Jerk Score of the Angular Velocity about Medio-Lateral Axis during the Sit-to-Walk Transition
Normalised Jerk Score of the Angular Velocity about Vertical Axis during the Sit-to-Walk Transition
Range Anterior-Posterior Acceleration during the Turn-to-Sit Transition [m/s^2]
Range Medio-Lateral Acceleration during the Turn-to-Sit Transition [m/s^2]
Range Vertical Acceleration during the Turn-to-Sit Transition [m/s^2]
Root Mean Square of the Anterior-Posterior Acceleration during the Turn-to-Sit Transition [m/s^2]
Root Mean Square of the Medio-Lateral Acceleration during the Turn-to-Sit Transition [m/s^2]
Root Mean Square of the Vertical Acceleration during the Turn-to-Sit Transition [m/s^2]

Jerk Score Anterior-Posterior Acceleration during the Turn-to-Sit Transition [m]
Jerk Score Medio-Lateral Acceleration during the Turn-to-Sit Transition [m]
Jerk Score Vertical Acceleration during the Turn-to-Sit Transition [m]
Range of the Angular Velocity about Anterior-Posterior Axis during the Turn-to-Sit Transition [°/s]
Range of the Angular Velocity about Medio-Lateral Axis during the Turn-to-Sit Transition [°/s]
Range of the Angular Velocity about Vertical Axis during the Turn-to-Sit Transition [°/s]
Root Mean Square of the Angular Velocity about Anterior-Posterior Axis during the Turn-to-Sit Transition [°/s]
Root Mean Square of the Angular Velocity about Medio-Lateral Axis during the Turn-to-Sit Transition [°/s]
Root Mean Square of the Angular Velocity about Vertical Axis during the Turn-to-Sit Transition [°/s]
Normalised Jerk Score of the Angular Velocity about Anterior-Posterior Axis during the Turn-to-Sit Transition
Normalised Jerk Score of the Angular Velocity about Medio-Lateral Axis during the Turn-to-Sit Transition
Normalised Jerk Score of the Angular Velocity about Vertical Axis during the Turn-to-Sit Transition
Turning Angle 180° Turn [°]
Turning Angle of the Turn-to-Sit Phase [°]
Mean Angular Velocity of the 180° Turn [°/s]
Mean Angular Velocity of the Turn-to-Sit Phase [°/s]
Peak Angular Velocity of the 180° Turn [°/s]
Peak Angular Velocity of the Turn-to-Sit Phase [°/s]
Normalised Jerk Score of the 180° Turn
Normalised Jerk Score of the Turn-to-Sit Phase
Walk Duration [s]
Gait Speed [m/s]
Number of Steps in the Walk Phase (not including turns)
Mean Step Length [m]
Mean Step Duration [s]
Step Duration Standard Deviation [s]
Step Duration Coefficient of Variation [%]
Mean Phase Differences [°]
Phase Differences Standard Deviation [°]
Mean Phase [°]
Phase Standard Deviation [°]
Phase Coefficient of Variation [%]
Phase Coordination Index [%]
Time-Normalised Jerk Score in the Anterior-Posterior direction [m]
Time-Normalised Jerk Score in the Medio-Lateral direction [m]
Time-Normalised Jerk Score in the Vertical direction [m]
Normalised Jerk Score in the Anterior-Posterior direction
Harmonic Ratio in the Anterior-Posterior direction
Harmonic Ratio in the Medio-Lateral direction
Harmonic Ratio in the Vertical direction

Step Regularity in the Anterior-Posterior Direction [%]
Step Regularity in the Medio-Lateral Direction [%]
Step Regularity in the Vertical Direction [%]
Stride Regularity in the Anterior-Posterior Direction [%]
Stride Regularity in the Medio-Lateral Direction [%]
Stride Regularity in the Vertical Direction [%]
Cadence [steps/min]
Gait Simmetry in the Anterior-Posterior Direction
Gait Simmetry in the Medio-Lateral Direction
Gait Simmetry in the Vertical Direction
Range Anterior-Posterior Acceleration during the Walk Phase [m/s^2]
Range Medio-Lateral Acceleration during the Walk Phase [m/s^2]
Range Vertical Acceleration during the Walk Phase [m/s^2]
Root Mean Square of the Anterior-Posterior Acceleration during the Walk Phase [m/s^2]
Root Mean Square of the Medio-Lateral Acceleration during the Walk Phase [m/s^2]
Root Mean Square of the Vertical Acceleration during the Walk Phase [m/s^2]
Range of the Angular Velocity about Anterior-Posterior Axis during the Walk Phase [°/s]
Range of the Angular Velocity about Medio-Lateral Axis during the Walk Phase [°/s]
Range of the Angular Velocity about Vertical Axis during the Walk Phase [°/s]
Root Mean Square of the Angular Velocity about Anterior-Posterior Axis during the Walk Phase [°/s]
Root Mean Square of the Angular Velocity about Medio-Lateral Axis during the Walk Phase [°/s]
Root Mean Square of the Angular Velocity about Vertical Axis during the Walk Phase [°/s]
Number of Steps in the 180° Turn
Power of the Vertical Push Off in the Sit-to-Walk Transition [Nm]
Jerk Ratio of the Antero-Posterior Direction in the Walk Phase
Jerk Ratio of the Medio-Lateral Direction in the Walk Phase

S2 List of clinical features

Table S2: Full list of features obtained from clinical assessments. It is worth noting that both TUG and 10-meter walking test were repeated five times per subject and each repetition is considered independently.

Clinical Features
Duration TUG (mean score over 5 repetitions)
Walking speed (10MWT, mean score over 5 repetitions)
MiniBest
POMAB
Conley
FES-I
FIM motor
FIM
